# Real-world outcomes of reduced-dose versus standard-dose antibody drug conjugates in metastatic breast cancer: a retrospective cohort study

**DOI:** 10.1007/s10549-025-07891-4

**Published:** 2026-01-20

**Authors:** Nickolas Stabellini, Jasskiran Kaur, Cynthia Owusu, Bahar Moftakhar, Takae Mizukami, Sonia D. de Oliveira, Alberto J. Montero

**Affiliations:** 1 Department of Hematology-Oncology, University Hospitals Seidman Cancer Center, 11100 Euclid Ave, Cleveland, OH 44106 USA; 2 Case Western Reserve University, Cleveland, OH USA; 3 Immunomonitoring Laboratory, Cleveland Clinic, Center for Immunotherapy & Precision Immuno-Oncology, Cleveland, OH USA; 4 Division of Cardiology, Department of Medicine, Medical College of Georgia at Augusta University, Augusta, GA USA

**Keywords:** Metastatic breast cancer, Reduced dose, Survival, Sacituzumab govitecan, Trastuzumab deruxtecan, Antibody–drug conjugate

## Abstract

**Purpose:**

To evaluate whether reduced doses (RD) of trastuzumab deruxtecan (T-DXd) or sacituzumab govitecan (SG) provide similar outcomes to the approved standard doses (SD) in metastatic breast cancer (mBC).

**Methods:**

This retrospective cohort included mBC patients receiving at least one cycle of SG (April 2021–May 2024) or T-DXd (February 2020–December 2024). Primary outcomes were progression-free survival (PFS) and overall survival (OS). Kaplan–Meier curves and Log-Rank tests estimated and compared PFS and OS from treatment initiation. Subgroup analyses were performed by HER2 and hormone receptor status.

**Results:**

48 patients received SG (24 RD vs. 24 SD) and 66 received T-DXd (29 RD vs. 37 SD). Median PFS for SG was 3 months in both SD (95% CI, 2–10) and RD (95% CI, 2–8; p = 0.8). Median OS for SG was 10 months (95% CI, 7–13) for SD and 11 months (95% CI, 5–30; p = 0.4) for RD. For T-DXd, median PFS was 10.4 months for SD (95% CI, 7.0–14.5) and 11.2 months for RD (95% CI, 5.4–31.1; p = 0.8), while median OS was 18.3 months (95% CI, 13.9–NA) for SD and 28.1 months (95% CI, 18.2–NA; p = 0.9) for RD. Overall response rates were similar between patients receiving RD and SD SG or T-DXd.

**Conclusions:**

This real-world data suggest RD of SG or T-DXd achieve outcomes comparable to SD, supporting prospective evaluation of lower-dose regimens.

## Introduction

Breast cancer remains one of the most prevalent cancers with an estimated 316,950 new cases in the United States (U.S.) alone expected in 2025 [1]. Metastatic breast cancer (mBC) remains incurable; however, the development of novel therapies such as antibody–drug conjugates (ADC) have transformed the treatment of mBC [2, 3]. ADCs are targeted treatments which combine a monoclonal antibody (mAb), and a cytotoxic chemotherapy agent via a linker to enable selective delivery of potent cytotoxic payload to target cancer cells [4]. As of June 2025, 19 ADCs have been approved globally for the treatment of various cancer types, including trastuzumab deruxtecan (T-DXd) and sacituzumab govitecan (SG) for treatment of advanced breast cancer patients [5–7]. T-DXd is a human epidermal growth factor 2 (HER2) directed antibody–drug conjugate currently approved for HER2 + and HER2 low or ultralow breast cancer [5]. In addition to breast cancer, T-DXd is also approved for HER2-mutant non-small-cell lung cancer, HER2-positive gastric or gastroesophageal junction adenocarcinoma, and any unresectable or metastatic HER2-positive (IHC 3 +) solid tumors [8–10]. SG is a Trop-2–directed ADC approved for unresectable, locally advanced or metastatic triple-negative breast cancer (TNBC) following ≥ 2 prior systemic therapies and pretreated hormone receptor (HR) + / HER2- mBC refractory to endocrine-based therapy [6]. Compared to physicians’ choice chemotherapy, T-DXd and SG significantly prolonged both progression-free survival and overall survival in phase III clinical trials [5, 6].

Cancer drug dosing has traditionally been based on the maximum tolerated dose (MTD) identified in phase 1 trials, which then in turn becomes the recommended phase II dose (RP2D) [11–13]. This method of dose determination operates on the assumption that toxicity can serve as a surrogate for cytotoxic drug efficacy [11–13]. While chemotherapy dose intensity may lead to improved outcomes in the non-metastatic setting, several trials have consistently demonstrated in mBC that dose intensity is not associated with improved survival outcomes [14]. For example, in a randomized trial evaluating three different doses of docetaxel no survival advantage was observed for 100 mg/m^2^ vs 60 mg/m^2^ of docetaxel, but greater dose intensity was associated with increased toxicities [15]. More recently, the well-designed and pragmatic X-7/7 trial demonstrated that there was no advantage to the higher Federal Drug Administration (FDA) approved dose of capecitabine in mBC, and that a lower fixed dose given on a novel one week on one week off schedule had similar efficacy with much less toxicity [16]. Interestingly, real-world retrospective data with fixed dose capecitabine had indicated that reducing dose intensity would not  reduce the clinical activity of capecitabine in advance of the randomized prospective trial [17].

While the efficacy and safety of ADC has been well-studied in controlled trials, these studies lack data on multiple dose levels hindering our analyses of long‐term clinical safety, efficacy, and real-world applications [18]. Theoretically, ADCs with their targeted delivery of chemotherapy as a drug class should have a more favorable therapeutic index than conventional cytotoxic chemotherapy [4]. However, rare but clinically significant, toxicities such as interstitial lung disease/pneumonitis and heart failure exist [19–22]. Toxicities significantly impact patient quality of life (QoL) and desire to continue with treatment [23, 24]. Given that lower doses of both docetaxel and capecitabine in mBC have shown similar efficacy with significantly less toxicity compared to higher doses, it is reasonable to expect that the same would apply to ADCs [25, 26]. We hypothesized that slightly lower doses of T-DXd and SG may offer comparable efficacy and less toxicity in treating mBC than the FDA-approved dose. Given the absence of prospective randomized trials that address the impact of either T-DXd or SD dose reduction on clinical outcomes in mBC real-world data are needed. Our study aimed to retrospectively address this knowledge gap.

## Methods

### Study setting

The study setting was University Hospitals Seidman Cancer Center (UHSCC), a large hybrid tertiary academic-community practice located in Northeast Ohio, U.S. The institution serves urban, suburban, and rural areas and comprises an integrated network of more than 23 hospitals, more than 50 health centers and outpatient facilities, and more than 200 physician offices across 16 counties throughout the region [27–29].

### Data source and covariates

De-identified real-world data were manually extracted from electronic health records (EHRs). The covariates captured included age, age at diagnosis, race, TNM stage at diagnosis, date of diagnosis, date of metastatic diagnosis, days from diagnosis to metastatic recurrence, number of different sites of disease, metastasis location, chemotherapy, metastasis at baseline, metastatic lines of therapy, T-DXd initial dose, T-DXd start date, T-DXd last dose date, SG initial dose, SG start date, SG last dose date, reason for dose reduction, subsequent dose reductions, number of treatments (total number of times the drug was administered from start to last recorded), date of progression, toxicities, hospitalizations from toxicity, treatment response, hormone receptor status, BRCA mutation, vital status, last follow-up date, date of death, and cause of death.

### Study design and population

**Fig. 1:**
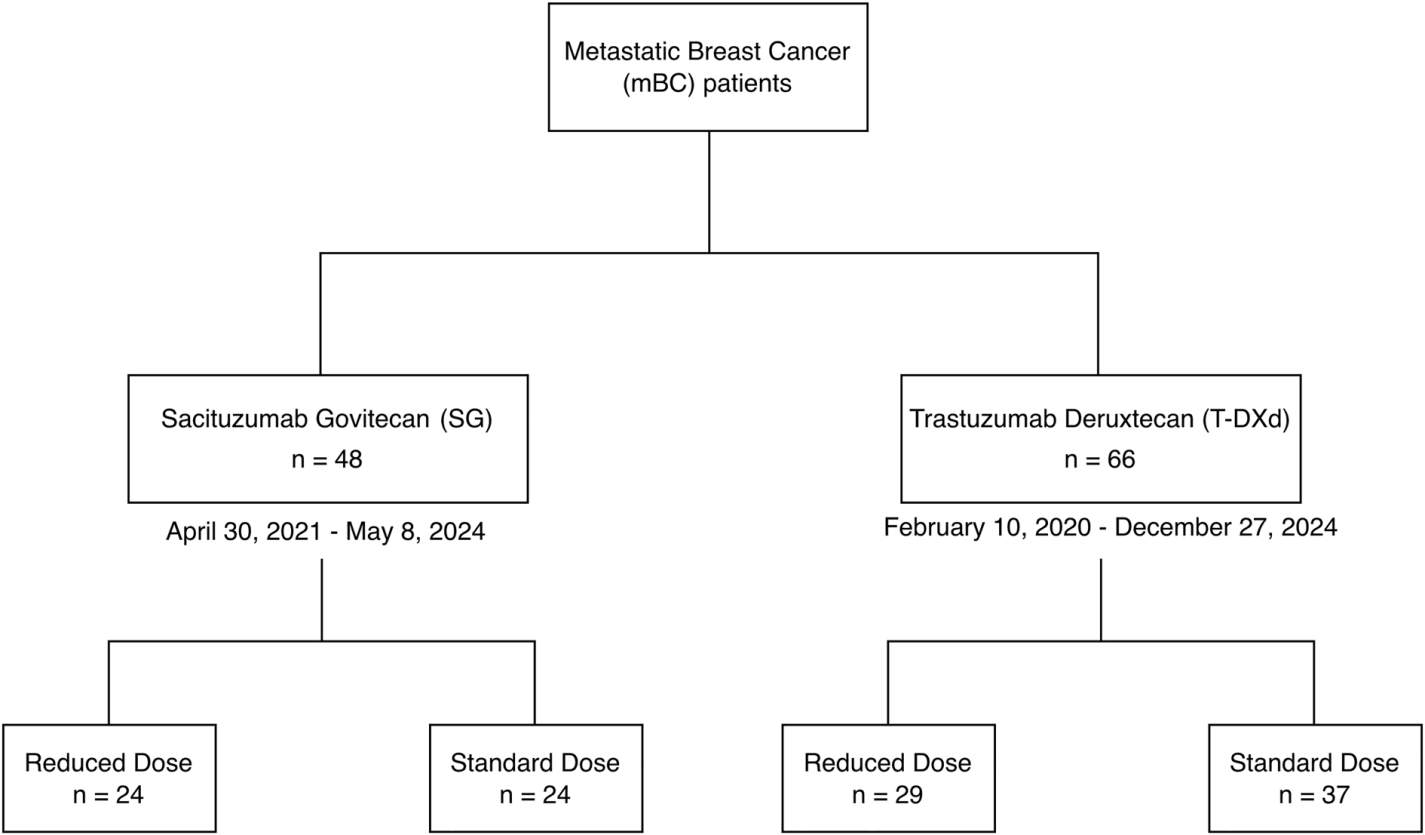
Study design and cohort distribution

This retrospective cohort study (Fig. 1) included mBC patients who received at least one cycle of SG between April 30, 2021 and May 8, 2024, and mBC patients who received at least one cycle of T-DXd between February 10, 2020 and December 27, 2024. Patients were included if they had received at least one cycle of treatment to minimize selection bias, as excluding those who discontinued therapy after one or two cycles due to treatment-related toxicities could artificially improve the apparent clinical outcomes.

### Outcomes

The primary outcomes were real-world progression-free survival (PFS) and overall survival (OS), and the secondary outcome was overall response rate (ORR).

### Statistical analysis

Categorical covariates were summarized using absolute numbers and percentages and statistically compared using the Pearson’s Chi-squared (χ^2^). Continuous covariates had their distribution assessed using histograms and the Kolmogorov–Smirnov test. If normally distributed, continuous covariates were summarized using mean and standard deviation and compared using the t-test. If non-normally distributed, continuous covariates were summarized using median and quartiles and compared using the Kruskal–Wallis test. Statistical comparisons considered patients receiving a reduced dose (RD) versus those receiving a standard-dose (SD) regimen.

The ORR was defined as the proportion of patients who achieved either a complete or partial response. ORR was calculated for each dose regimen and compared using χ^2^ test.

Kaplan–Meier methodology was used to estimate real-world PFS and OS from the date of treatment (SG or T-DXd) initiation, overall and by dose regimen. The log-rank test was applied to compare PFS and OS according to dose regimen. Subgroup analyses for the Kaplan–Meier and log-rank test were performed for SG in TNBC, ER + , and ER + /HER2-low patients, and for T-DXd in TNBC, HER2 + /ER-, HER2 + /ER + , and HER2-/ER + patients.

A p-value < 0.05 was considered statistically significant.

### Software

All analyses were performed in R (version 4.4.3) [30, 31].

## Results

### Sacituzumab govitecan (SG) cohort

Our study included 48 mBC patients who received SG (Table 1). The median age of these patients at SG initiation was 63 years (IQR 50–70), with a median age at initial breast cancer diagnosis of 54 years (IQR 42–64). Patients were followed for a median of 65 months (IQR 33.6–123.5) from their initial breast cancer diagnosis, 28.6 months (IQR 13.4–68.68) from the mBC diagnosis, and 10.5 months (IQR 5.33–15.38) from the start of first SG treatment.

**Table 1:** Demographic characteristics of the overall cohort and stratified by standard versus reduced dose regimens for the sacituzumab govitecan (SG) cohort

Characteristic	Overall	Reduced Dose	Standard Dose	p-value
	n = 48	n = 24 (50%)	n = 24 (50%)	
Age in years—median (IQR)	64 (50–70)	65 (60–72)	61 (39–66)	0.08
Age at diagnosis in years—median (IQR)	54 (41.7–63.2)	56 (50–64)	49 (38–60)	0.06
Race—n (%)				
Black	14 (29.2)	6 (25)	8 (33)	0.72
White	31 (64.6)	16 (67)	15 (63)	
Hispanic	2 (4.2)	1 (4.2)	1 (4.2)	
Asian	1 (2.1)	1 (4.2)	0	
Stage at Initial Diagnosis—n (%)				
I-II	22 (45.8)	14 (58)	8 (33)	*0.03*
III	19 (39.6)	5 (21)	14 (58)	
IV	7 (14.6)	5 (21)	2 (8.3)	
HER2 IHC—n (%)				
0	19 (39.6)	10 (42)	9 (38)	0.81
1	14 (29.2)	6 (25)	8 (33)	
2	15 (31.2)	8 (33)	7 (29)	
ER +—n (%)	14 (29.2)	9 (37.5)	5 (21)	0.34
TNBC—n (%)	34 (70.8)	15 (62.5)	19 (79.2)	0.34
BRCA 1/2 mutated—n (%)	1 (2.1)	0	1 (4.2)	-
Prior neo/adjuvant chemo—n (%)	22 (45.8)	18 (75)	4 (17)	< 0.001
Prior KN-522 regimen—n (%)	11 (22.9)	0	11 (46)	< *0.001*
Prior anthracyclines/taxanes (no pembro)—n (%)	23 (47.9)	14 (58)	9 (38)	0.24
Prior Taxanes Only—n (%)	4 (8.3)	4 (17)	0	0.11
Prior Lines Therapy in Metastatic Setting—median (IQR)	2 (1–4)	3 (2–4)	1 (0–2)	*0.002*
For ER + , prior CDK4/6—n (%)	14 (29.2)	9 (38)	5 (21)	0.34
Prior PD-1 mAB in metastatic setting—n (%)	21 (43.8)	12 (50)	9 (38)	0.56
Prior TDxD—n (%)	4 (8.3)	3 (12.5)	1 (4.2)	0.60
Visceral mets at Baseline—n (%)	40 (83.3)	21 (87.5)	19 (79)	0.69
Number of different sites of disease, median (IQR)	3 (2–3)	3 (3–3)	3 (2–4)	0.7
Brain Mets—n (%)	11 (22.9)	5 (21)	6 (25)	1
Number of treatments – median (IQR)	9.5 (6–26)	10 (6–23)	9.5 (7–22)	0.92

The cohort was predominantly White (64.6%). Most patients were initially diagnosed at TNM stages I or II (45.8%), and the majority (70.8%) had TNBC.

### Trastuzumab deruxtecan (T-DXd) cohort

We analyzed data from 66 mBC patients who received T-DXd (Table 2). Their median age at time of receiving T-DXd was 67 years (IQR 62.0 to 74.7), with a median age at diagnosis of 57.5 years (IQR 47.0 to 64.0). The median follow-up was 111 months (IQR 65.2 to 174.2) from initial breast cancer diagnosis, 62.7 months (IQR 30.1–104.2) from the mBC diagnosis, and 16.8 months (IQR 8.8–26.5 months) from initial T-DXd.

**Table 2:** Demographic characteristics of the overall cohort and stratified by standard versus reduced dose regimens for the trastuzumab deruxtecan (T-DXd) cohort

Characteristic	Overall	Standard dose	Reduced dose	p-value
	n = 66	n = 37	n = 29	
Age in years—median (IQR)	67 (62.0–74.7)	67.0 (57.0–73.0)	69.0 (65.0–79.0)	*0.04*
Age at diagnosis in years—median (IQR)	57.5 (47.0–64.0)	52.0 (43.0–64.0)	60.0 (54.0–67.0)	0.07
Race—n (%)				
Black	17 (25.8)	9 (24.3)	8 (27.6)	0.93
White	47 (71.2)	27 (73.0)	20 (69.0)	
Hispanic	0	0	0	
Asian	0	0	0	
Other	2 (3.0)	1 (2.7)	1 (3.4)	
Stage at Initial Diagnosis—n (%)				
I-II	19 (34.5)	12 (38.7)	7 (29.2)	0.74
III	12 (21.8)	6 (19.4)	6 (25.0)	
IV	24 (43.6)	13 (41.9)	11 (45.8)	
HER2 +—n (%)	27 (40.9)	16 (43.2)	11 (37.9)	0.85
ER +—n (%)	47 (71.2)	24 (64.9)	23 (79.3)	0.31
HER2 + /ER-	10 (15.1)	7 (18.9)	3 (10.3)	0.49
HER2 + /ER +	17 (25.7)	9 (24.3)	8 (27.5)	0.78
HER2 low/ER +	30 (45.4)	15 (40.5)	15 (51.7)	0.45
TNBC—n (%)	7 (10.6)	5 (13.5)	2 (6.9)	0.64
BRCA 1/2 mutated—n (%)	2 (3)	0	2 (6.9)	-
No prior neo/adjuvant chemo—n (%)	9 (13.6)	6 (16.2)	3 (10.3)	0.78
Visceral mets at Baseline—n (%)	49 (74.2)	26 (70.2)	23 (79.3)	0.71
Number of different sites of disease, median (IQR)	3.0 (2.0–4.0)	3.0 (2.0–4.0)	3.0 (2.0–3.0)	0.10
Brain Mets—n (%)	18 (27.7)	14 (37.8)	4 (13.8)	0.05
Pneumonitis—n (%)	3 (4.5)	3 (8.1)	0	-
Number of treatments – median (IQR)	11 (6–26)	14 (4–36)	11 (8–16)	0.54

The majority of patients were White (71.2%). Most (43.6%) were diagnosed at TNM stage IV, and the cohort was predominantly ER + (71.2%), with 40.9% being diagnosed with HER2 + disease.

### Treatment regimen and characteristics

In the SG cohort (Table 1), 45.8% of patients had received prior neo/adjuvant chemotherapy, 22.9% had the KN-522 regimen, and 47.9% had prior anthracyclines/taxanes [32]. In addition, 44% had prior PD-1 mAb in the metastatic setting.

Of the 48 SG patients, 24 (50%) received an initial RD of SG at 8 mg/kg; the other 50% received an initial SD of SG at 10 mg/kg. Two patients (1 RD, 1 SD) received only one cycle of treatment (two administrations). The median time on SG was 3.5 months (IQR 1.5–7.2) for RD and 3.3 months (IQR 2.3–8.1) for SD (p = 0.42). When comparing these two groups (Table 1), the RD group had a higher rate of TNM stage IV at diagnosis (21% vs. 8.3%, p = 0.03). Patients who received the SD SG were more likely to have received prior neo/adjuvant chemotherapy (75% vs. 17%, p < 0.001). Among TNBC patients, those who received SD SG were less likely to have KN-522 regimen (0% vs. 46%, p < 0.001). Furthermore, the RD group had a higher median of prior lines of therapy in the metastatic setting (3 [IQR 2–4] vs 1 [IQR 0–2], p = 0.002). From cycle 1 day 8 onward, subsequent dose reductions occurred in 9 of 24 RD patients (37.5%) and 13 of 24 SD patients (54.2%), while the remaining patients in each group maintained their initial dose. The difference in dose reduction rates between groups was not statistically significant (p = 0.38). Myeloid growth factor use was significantly higher in patients receiving an initial SD of SG (58% vs. 0; p < 0.01). Moreover, we also observed that SD patients had higher rates of hospitalization due to SG toxicity compared to RD (20.8% vs. 4.3%, p = 0.20).

For the T-DXd cohort (Table 2), 13.6% of patients had no prior neo/adjuvant chemotherapy. Of the 66 T-DXd patients, 29 (43.9%) received an initial RD at 4.4 mg/kg. Compared to the SD group (Table 2), those started on the RD of T-DXd were older (median age of 69 years [IQR 65.0–79.0] vs. 67 years [IQR 57.0–73.0], p = 0.04). The median time on T-DXd was 9.8 months (IQR 2.0–18.8) for RD and 7.8 months (IQR 4.5–13.2) for SD (p = 0.88). Nine patients (4 RD, 5 SD) received only 1–2 cycles of treatment. From cycle 2 onward, subsequent dose reductions occurred in none of the RD patients and 5 of 37 SD patients (13.5%), while the remaining patients in each group maintained their initial dose. The difference in dose reduction rates between groups was not statistically significant (p = 0.11).

No dose escalations were observed among patients who initiated treatment at RD of SG or T-DXd. Among patients who started at SD and subsequently required dose reductions, treatment was continued at the RD level, with no attempts at dose re-escalation.

### Survival

**Fig. 2:**
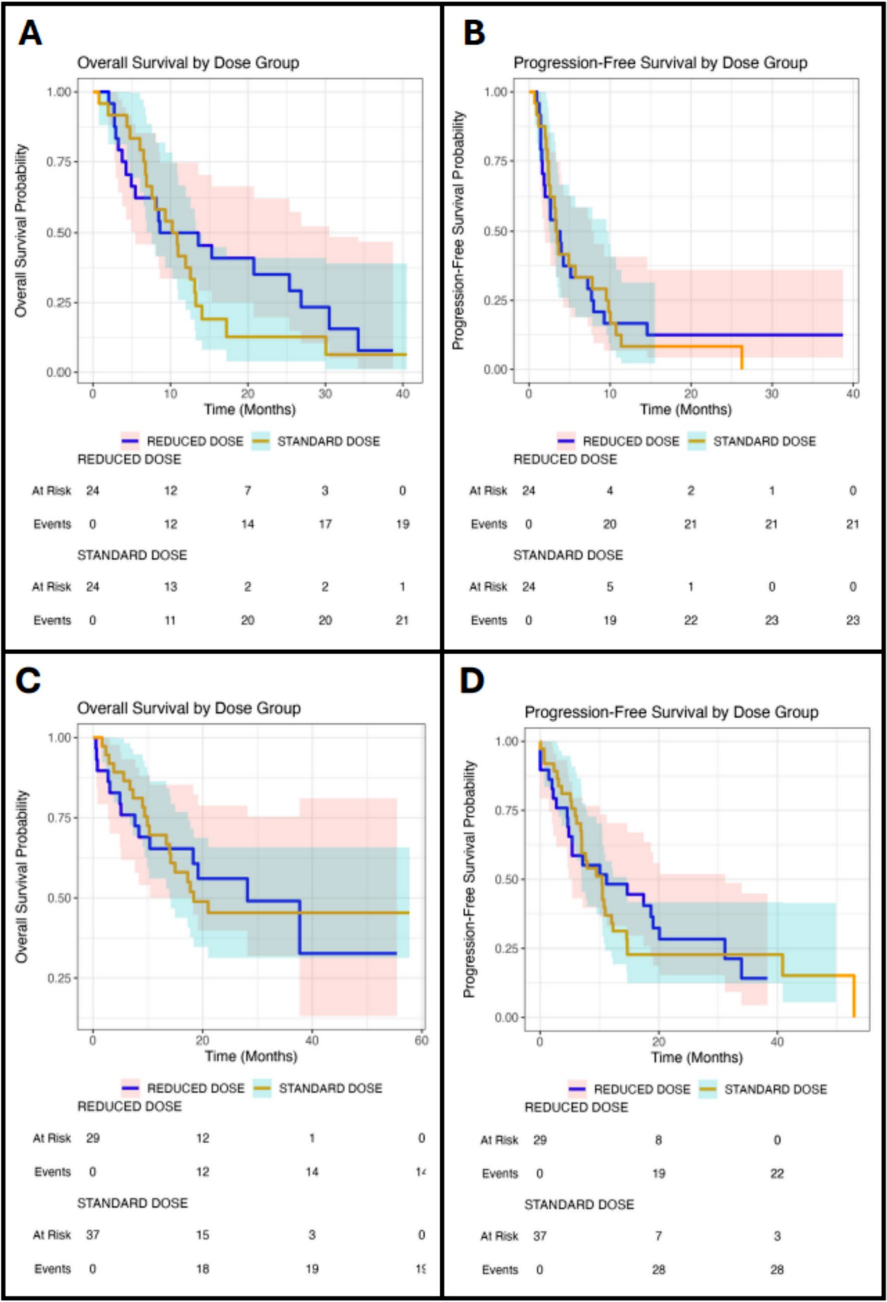
Kaplan–Meier curves for real-world overall survival (OS) and progression-free survival (PFS) comparing standard-dose (yellow) and reduced dose (blue) treatments: **A** OS for sacituzumab govitecan, **B** PFS for sacituzumab govitecan, **C** OS for trastuzumab deruxtecan, and **D** PFS for trastuzumab deruxtecan

At the last follow-up, 16.7% of SG patients were alive. The median PFS from the time of SG initiation was 3 months for both SD (95% CI, 2–10) and RD (95% CI, 2–8, showing no statistical difference between the two doses (p = 0.8; Fig. 2). Similarly, the median survival (Table 3) did not significantly differ between SD (10 months; 95% CI 7–13) and RD (11 months; 95% CI 5–30; p = 0.4; Fig. 2) groups. From initial breast cancer diagnosis, the median survival was 68 months (95% CI 49–123), and 29 months (95% CI 19–68) from the date of initial mBC diagnosis. The 12-month and 24-month survival rates for the overall SG cohort and by dose regimen are shown in Table 4.

**Table 3:** Median real-world progression-free survival (PFS) and median real-world survival from treatment initiation for the overall cohort, stratified by dose regimen across treatment cohorts and subgroups of interest

	Overall	Standard dose	Reduced dose	p-value
SG	n = 48	n = 24	n = 24	
Median PFS (95% CI)	3.4 (2.6–5.7)	3.4 (2.6–9.5)	3.5 (1.9–7.7)	0.9
Median Survival (95% CI)	10.6 (8.0–14.1)	10.6 (7.6–13.3)	11.1 (5.4–30.5)	0.4
TNBC (n = 34)				
Median PFS (95% CI)	3.2 (2.6–7.8)	3.2 (2.4–9.8)	3.9 (2.6-NA)	0.1
Median Survival (95% CI)	11.5 (8.6–25.4)	10.9 (8.0–17.2)	25.4 (8.4-NA)	0.2
ER + (n = 14)				
Median PFS (95% CI)	3.5 (1.6–10.7)	5.7 (4.9-NA)	1.6 (1.4-NA)	0.09
Median Survival (95% CI)	6.4 (4.3-NA)	6.7 (6.0-NA)	4.9 (3.7-NA)	0.8
ER + /HER2 low (n = 14)				
Median PFS (95% CI)	3.5 (1.6–10.7)	5.7 (4.9-NA)	1.6 (1.4-NA)	0.09
Median Survival (95% CI)	6.4 (4.3-NA)	6.7 (6.0-NA)	4.9 (3.7-NA)	0.8
T-DXd	n = 66	n = 37	n = 29	
Median PFS (95% CI)	10.4 (7.0–14.6)	10.4 (7.0–14.5)	11.2 (5.4–31.1)	0.8
Median Survival (95% CI)	21 (14.9-NA)	18.4 (14.0-NA)	28.2 (18.3-NA)	1
TNBC (n = 7)				
Median PFS (95% CI)	5.9 (2.8-NA)	5.9 (2.8-NA)	12.0 (5.4-NA)	0.5
Median Survival (95% CI)	9.9 (6.7-NA)	9.9 (6.7-NA)	-	0.1
ER + (n = 47)				
Median PFS (95% CI)	10.4 (7.0–17.4)	9.9 (7.0–14.7)	11.2 (4.6-NA)	0.9
Median Survival (95% CI)	21.0 (14.9-NA)	NA (14.1-NA)	19.2 (8.3-NA)	0.5
HER2 + (n = 27)				
Median PFS (95% CI)	14.7 (9.4-NA)	11.5 (7.0-NA)	20.1 (7.1-NA)	0.5
Median Survival (95% CI)	37.7 (18.3-NA)	NA (17.3-NA)	28.2 (18.3-NA)	0.4
HER2 low/ER + (n = 30)				
Median PFS (95% CI)	7.8 (5.2–12.3)	7.6 (6.1–12.3)	10.0 (2.2-NA)	0.5
Median Survival (95% CI)	14.2 (9.0-NA)	14.2 (9.4-NA)	19.2 (4.9-NA)	0.9
HER2 + /ER- (n = 10)				
Median PFS (95% CI)	11.5 (5.1-NA)	10.9 (5.1-NA)	NA (2-NA)	0.5
Median Survival (95% CI)	NA (10.3-NA)	NA (10.2-NA)	NA (3.0-NA)	0.94
HER2 + /ER- (n = 17)				
Median PFS (95% CI)	14.7 (9.4-NA)	14.7 (9.4-NA)	17.3 (7.1-NA)	0.69
Median Survival (95% CI)	NA (37.7-NA)	NA (NA-NA)	37.7 (18.2-NA)	0.11

**Table 4:** 12- and 24-month survival rates from treatment initiation for the overall cohort, stratified by dose regimen across treatment cohorts and subgroups of interest

Survival rate, % (95% CI)	Overall	Standard dose	Reduced dose
SG	n = 48	n = 24	n = 24
12-month	43.7 (31.6–60.2)	37.5 (22.3–62.8)	50.0 (33.5–74.6)
24-month	24 (14.0–41.1)	12.7 (3.9–40.9)	35.1 (19.8–62.20
TNBC (n = 34)			
12-month	46.9 (32.7–67.1)	36.8 (20.4–66.4)	60.0 (39.7–90.7)
24-month	29.9 (17.5–51.2)	12.6 (3.5–44.5)	52.5 (32.3–85.6)
ER + (n = 14)			
12-month	35.7 (17.7–72.1)	40 (13.7–100.0)	33.3 (13.2–84.0)
24-month	–	–	–
ER + /HER2 low (n = 29)			
12-month	35.7 (17.7–72.1)	40 (13.7–100.0)	33.3 (13.2–84.0)
24-month	–	–	–
T-DXd	n = 66	n = 37	n = 29
12-month	67.8 (57.3–80.2)	69.5 (56.0–86.3)	65.3 (50.0–85.2)
24-month	49.6 (38.3–64.2)	45.3 (31.2–65.7)	56.0 (39.8–78.7)
TNBC (n = 7)			
12-month	47.6 (18.8–100.0)	30.0 (6.3–100.0)	100.0 (100.0–100.0)
24-month	–	–	–
ER + (n = 47)			
12-month	70.0 (58.0–84.5)	78.7 (63.8–97.1)	60.9 (43.9–84.5)
24-month	49.5 (36.4–67.3)	50.9 (33.6–77.1)	48.7 (31.0–76.4)
HER2 + or PR + (n = 48)			
12-month	68.5 (56.6–83.1)	75.4 (60.1–94.6)	60.9 (43.9–84.5)
24-month	48.4 (35.5–66.1)	48.7 (31.9–74.5)	48.7 (31.0–76.4)
HER2 + (n = 27)			
12-month	77.4 (63.1–95.1)	80.8 (63.4–100.0)	72.7 (50.6–100.0)
24-month	65.2 (49.1–86.5)	67.3 (47.4–95.6)	62.3 (38.9–99.9)
HER2 low/ER + (n = 30)			
12-month	60.0 (44.8–80.4)	66.7 (46.6–95.3)	53.3 (33.2–85.6)
24-month	34.4 (20.0–59.2)	29.2 (12.3–69.0)	42.7 (22.4–81.3)
HER2 + /ER- (n = 10)			
12-month	70.0 (46.7–100.0)	71.4 (44.7–100.0)	66.7 (30.0–100.0)
24-month	60.0 (36.2–99.5)	57.1 (30.1–100.0)	66.7 (30.0–100.0)
HER2 + /ER + (n = 17)			
12-month	87.8 (73.4–100.0)	100.0 (0–100.0)	75 (50.3–100.0)
24-month	74.3 (55.4–99.7)	87.5 (11.7–67.3)	60 (33.1–100.0)

Among TNBC patients treated with SG, the median PFS was 3.4 months (95% CI, 2.6–7.8) and the median OS was 11.5 months (95% CI, 8.6–25.4), with no statistically significant differences between RD and SD for either outcome (PFS p = 0.10; OS p = 0.20; Table 3). Similarly, in ER + /HER2 − mBC patients, the median PFS was 3.5 months (95% CI, 1.6–10.7) and the median OS was 6.42 months (95% CI, 4.3–NA), with no statistically significant differences between RD and SD (PFS p = 0.09; OS p = 0.80; Table 3).

At last follow-up, 50% of T-DXd patients were alive. The median PFS from the time of T-DXd initiation was 10.4 months for SD (95% CI 7.0–14.5) and 11.2 months for RD (95% CI 5.4–31.1), indicating no statistically observable significant difference between both dosing regimens (p = 0.8; Fig. 2). Similarly, the median OS was not statistically different between SD (18.3 months; 95% CI 13.9–NA) and RD (28.1 months; 95% CI 18.2–NA; p = 0.9; Fig. 2) groups. The overall median OS from the time of initial T-DXd initiation was 21 months (95% CI 14.9–15). The 12-month and 24-month survival rates for the overall T-DXd cohort and by dose regimen are shown in Table 4.

For HER2 + mBC patients, the median PFS from date of T-DXd initiation was 14.6 months (95% CI 9.4-NA), without any observable statistically significant differences between RD and SD (p = 0.5; Table 3). The median OS from date of T-DXd initiation was 37.7 months (95% CI 18.2-NA). For ER + /HER2- mBC patients, the overall median PFS was 7.8 months (95% CI 5.2- 12.2), without any significant statistical differences observed between RD and SD (p = 0.5; Table 3), while the median OS for TDxD was 14.1 months (95% CI 9.0-NA). For HER2 + /ER- mBC patients, the median PFS was 11.5 months (95% CI: 5.1–NA) and the 24-month survival rate was 60.0% (95% CI: 36.2–99.5). For HER2 + /ER + mBC patients, the median PFS was 14.7 months (95% CI: 9.4–NA) with a 12-month survival rate of 87.8% (95% CI: 73.4–100.0). In all these subgroups, no statistically significant differences were observed between RD and SD for either PFS or OS (Table 3).

### Real-world response rates

The real-world overall response rate (rwORR) for the entire SG cohort was 20.8%. RD SG had a rwORR of 25%, compared to 16.7% for SD SG (p = 0.72). For specific subtypes, the rwORR among TNBC patients was 23.5% (33.3% RD vs. 15.8% SD, p = 0.42) and 14.3% (11.1% RD vs. 20% SD, p = 1.00) in patients with ER + /HER2- mBC.

The observed rwORR for the entire T-DXd cohort was 16.7% (24.1% RD vs. 10.8% SD, p = 0.26). For patients with HER2 + mBC the observed rwORR was 18.5% (18.8% SD vs. 18.2% RD, p = 1.00). For ER + /HER2- mBC the rwORR was 15.6% (6.2% SD vs. 25% RD, p = 0.32).

## Discussion

This retrospective cohort study aimed to explore the impact of RD T-DXd or SG, two FDA-approved ADCs for the treatment of mBC. We hypothesized that RD SG and T-DXd would have comparable real-world outcomes to SD in mBC, based on prior randomized trials with cytotoxic chemotherapy showing similar clinical outcomes with lower-dose intensity. We analyzed two cohorts: 48 patients treated with SG (24 RD, 24 SD) and 66 patients treated with T-DXd (29 RD, 37 SD). Our results did not find any statistically significant differences in real-world OS, PFS, or rwORR when comparing RD and SD, either overall or by HER2 and hormone receptor status. Moreover, RD of SG was associated with lower rates of hospitalization and growth factor use.

The goal of systemic therapy in metastatic cancer is not is not to administer the highest dose by default, but rather the most effective dose that will be best tolerated by patients, maximizing their chances of successful treatment, while at the same time preserving QoL and minimizing harm [33, 34]. This aligns with the FDA’s Project Optimus, which aims to develop strategies for dose-finding and optimization by leveraging both nonclinical and clinical data, including randomized evaluations of multiple dose levels in clinical trials [35]. The randomized phase 3 E1193 trial compared doxorubicin, paclitaxel, or combination doxorubicin and paclitaxel plus granulocyte colony-stimulating factor as first-line therapy [36]. Patients receiving single-agent doxorubicin or paclitaxel were crossed over to the other agent at time of progression [36]. This well-designed trial demonstrated while the combination of doxorubicin and paclitaxel was associated with higher ORR and time to treatment failure compared to each drug alone, combination therapy did not improve either survival or QoL compared to sequential single-agent therapy [36]. Just as polychemotherapy does not necessarily translate to better outcomes in mBC, the dose intensification of single agent chemotherapy may not translate to better clinical outcomes.

In the current era of ADCs and targeted delivery of chemotherapy, the overarching therapeutic goal in mBC should be to administer drugs at doses that provide the greatest average benefit with the least potential for toxicities, thereby maximizing patient QoL [33, 34]. Dose reduction strategies for antibody–drug conjugates like SG and TDxD in mBC represent a promising approach to optimize the therapeutic index by maintaining clinical efficacy while minimizing treatment-limiting toxicities [37]. Clinical evidence suggests that these agents often require dose modifications due to adverse events such as neutropenia, diarrhea, and interstitial lung disease, which can compromise treatment adherence and QoL [19–24]. Emerging pharmacokinetic and pharmacodynamic data indicate that the steep dose–response relationship traditionally assumed for cytotoxic agents may not apply to ADCs, where the targeted delivery mechanism and prolonged tumor exposure through linker technology may allow for sustained antitumor activity at reduced doses [4, 38–42]. Furthermore, retrospective analyses and small prospective studies have suggested that reduced dose intensity of ADCs do not negatively impact PFS or ORR while at the same time reduce the potential for adverse toxicities, suggesting that a more conservative dosing approach could preserve long-term treatment options and improve patient outcomes without compromising oncologic benefit [43–47].

Our study helps to address this knowledge gap  of the impact of RD intensity of ADCs in mBC which poses a challenge to clinicians who must weigh the risks and benefits of dose reduction, particularly in physiologically vulnerable adults. Our results are consistent with previous published findings which have shown similar clinical outcomes with reduced dose intensity, which may be beneficial for treatment adherence and patient QoL [37, 44–47]. Attempts to maintain ADC dose intensity, such as the PRIMED trial which maintained SG dose at 10 mg/kg by giving primary granulocyte colony-stimulating factor prophylaxis runs counter to the clinical trial literature in mBC that has shown that dose de-intensification of chemotherapy may be associated with more favorable side effect profile, and at the same time maintaining similar efficacy as higher chemotherapy doses [48, 49]. In this regard, less may indeed be more and when it comes to ADC’s, and the time has come to clinically test this approach in mBC.

Our study has several limitations. First, the single-institution, retrospective real-world design may introduce selection bias, as the findings could reflect only the patient population seen at this institution. The retrospective nature also increases the risk of missing and/or inaccurate data points. Regarding toxicities, for example, data in the EHR were not systematically graded using the standardized Common Terminology Criteria for Adverse Events criteria, and clinical documentation may vary between providers and encounters. As a result, we were unable to provide a comprehensive overview or direct comparison of toxicities between the SD and RD groups. Treatment patterns and supportive care protocols unique to this institution and its providers may have influenced outcomes, limiting generalizability. Selection bias may arise from physician-driven decisions regarding dose reduction and patient management. The inclusion periods differed between cohorts (patients receiving T-DXd were included from February 2020, while those receiving SG were included from April 2021), which could introduce temporal bias. Outcomes such as PFS and OS were derived from EHR, and thus may not align precisely with clinical trial–defined endpoints. The results are based on univariable analysis, therefore, they were not adjusted for other factors (e.g., tumor characteristics) that could influence the outcomes. Furthermore, statistical analyses were limited to unadjusted comparisons and did not account for potential confounders through multivariable modeling. Finally, the relatively small sample size may limit statistical power, particularly in subgroup analyses.

In conclusion, this retrospective cohort real-world data provides foundational evidence supporting the hypothesis that a RD of SG or T-DXd offers similar outcomes to the current SD. Given the relationship between toxicity, QoL, and treatment adherence, this evidence, corroborated by prior findings in the ADC field, supports the prospective evaluation of dose-reduced SG or T-DXd as a potential dose optimization strategy in mBC. Such an approach could have a meaningful impact on clinical practice and quality of care. Future studies should focus on employing multicentric, prospective, randomized designs, adjusted for confounders and with larger sample sizes, to validate and expand upon the findings of our study.

The part of the results of this manuscript were presented as poster at: 1. 2025 ASCO Quality Care Symposium (DOI 10.1200/OP.2025.21.10_suppl.569) 2. 2024 San Antonio Breast Cancer Symposium (DOI 10.1158/1557-3265.SABCS24-P3-12-14).

## Data Availability

University Hospitals Seidman Cancer Center data has access restricted to researchers with institutional review board approval.

## References

[1] Siegel RL, Kratzer TB, Giaquinto AN, Sung H, Jemal A (2025) Cancer statistics, 2025. CA Cancer J Clin 75(1):10–45 39817679 10.3322/caac.21871 PMC11745215

[2] Riggio AI, Varley KE, Welm AL (2021) The lingering mysteries of metastatic recurrence in breast cancer. Br J Cancer 124(1):13–26 33239679 10.1038/s41416-020-01161-4 PMC7782773

[3] Davis AA, Hesse J, Pereira PMR, Ma CX (2025) Novel treatment approaches utilizing antibody-drug conjugates in breast cancer. Npj Breast Cancer 11(1):42 40360516 10.1038/s41523-025-00743-w PMC12075872

[4] Fu Z, Li S, Han S, Shi C, Zhang Y (2022) Antibody drug conjugate: the “biological missile” for targeted cancer therapy. Signal Transduct Target Ther 7(1):93 35318309 10.1038/s41392-022-00947-7 PMC8941077

[5] Modi S, Jacot W, Yamashita T, Sohn J, Vidal M, Tokunaga E et al (2022) Trastuzumab deruxtecan in previously treated HER2-low advanced breast cancer. N Engl J Med 387(1):9–20 35665782 10.1056/NEJMoa2203690 PMC10561652

[6] Bardia A, Hurvitz SA, Tolaney SM, Loirat D, Punie K, Oliveira M et al (2021) Sacituzumab govitecan in metastatic triple-negative breast cancer. N Engl J Med 384(16):1529–1541 33882206 10.1056/NEJMoa2028485

[7] Wu D, Yang K, He R, Yin R, Shui L (2025) Antibody-drug conjugates in cancer therapy: current advances and prospects for breakthroughs. Front Cell Dev Biol 8(13):1669592 10.3389/fcell.2025.1669592 PMC12540542 41133214

[8] Shitara K, Bang YJ, Iwasa S, Sugimoto N, Ryu MH, Sakai D et al (2020) Trastuzumab deruxtecan in previously treated HER2-positive gastric cancer. N Engl J Med 382(25):2419–2430 32469182 10.1056/NEJMoa2004413

[9] Meric-Bernstam F, Makker V, Oaknin A, Oh DY, Banerjee S, González-Martín A et al (2024) Efficacy and safety of Trastuzumab Deruxtecan in patients with HER2-expressing solid tumors: primary results from the DESTINY-PanTumor02 phase II trial. J Clin Oncol 42(1):47–58 37870536 10.1200/JCO.23.02005 PMC10730032

[10] Goto K, Goto Y, Kubo T, Ninomiya K, Kim SW, Planchard D et al (2023) Trastuzumab deruxtecan in patients with HER2-mutant metastatic non–small-cell lung cancer: primary results from the randomized, phase II DESTINY-Lung02 trial. J Clin Oncol 41(31):4852–4863 37694347 10.1200/JCO.23.01361 PMC10617843

[11] Le Tourneau C, Lee JJ, Siu LL (2009) Dose escalation methods in phase I cancer clinical trials. JNCI J Natl Cancer Inst 101(10):708–720 19436029 10.1093/jnci/djp079 PMC2684552

[12] Levit LA, Shah M, Ratain MJ, Garrett-Mayer E, Rahman A, Theoret M, Harvey RD (2025) Totality of the evidence: optimizing dosage selection strategies in oncology. J Clin Oncol 43(25):2827–2833 40706003 10.1200/JCO-25-00488 PMC12313202

[13] Sleijfer S, Wiemer E (2008) Dose selection in phase I studies: why we should always go for the top. J Clin Oncol 26(10):1576–1578 18332465 10.1200/JCO.2007.15.5192

[14] Loibl S, Skacel T, Nekljudova V, Lück HJ, Schwenkglenks M, Brodowicz T et al (2011) Evaluating the impact of relative total dose intensity (RTDI) on patients’ short and long-term outcome in taxane- and anthracycline-based chemotherapy of metastatic breast cancer- a pooled analysis. BMC Cancer 12(11):131 10.1186/1471-2407-11-131 PMC3083375 21486442

[15] Harvey V, Mouridsen H, Semiglazov V, Jakobsen E, Voznyi E, Robinson BA et al (2006) Phase III trial comparing three doses of docetaxel for second-line treatment of advanced breast cancer. J Clin Oncol Off J Am Soc Clin Oncol 24(31):4963–4970 10.1200/JCO.2005.05.0294 17033039

[16] Khan QJ, Bohnenkamp C, Monson TE, Phadnis MA, Clark L, Smith HE et al (2025) Randomized trial of fixed-dose capecitabine compared with standard-dose capecitabine in metastatic breast cancer: X-7/7 trial. JCO Oncol Adv 2(1):e2400068 39917581 10.1200/OA-24-00068 PMC11797229

[17] Ambros T, Zeichner SB, Zaravinos J, Montero AJ, Ahn E, Aruna M et al (2014) A retrospective study evaluating a fixed low dose capecitabine monotherapy in women with HER-2 negative metastatic breast cancer. Breast Cancer Res Treat 146(1):7–14 24899084 10.1007/s10549-014-3003-x

[18] Liao MZ, Lu D, Kågedal M, Miles D, Samineni D, Liu SN et al (2021) Model-informed therapeutic dose optimization strategies for antibody-drug conjugates in oncology: what can we learn from US food and drug administration-approved antibody-drug conjugates? Clin Pharmacol Ther 110(5):1216–1230 33899934 10.1002/cpt.2278 PMC8596428

[19] Nguyen TD, Bordeau BM, Balthasar JP (2023) Mechanisms of ADC toxicity and strategies to increase ADC tolerability. Cancers 15(3):713 36765668 10.3390/cancers15030713 PMC9913659

[20] Masters JC, Nickens DJ, Xuan D, Shazer RL, Amantea M (2018) Clinical toxicity of antibody drug conjugates: a meta-analysis of payloads. Invest New Drugs 36(1):121–135 29027591 10.1007/s10637-017-0520-6

[21] Maurier L, Chéné AL, Hulo P, Chen J, Sagan C, Pons-Tostivint E (2025) [Diffuse interstitial lung disease induced by antibody-drug conjugates]. Rev Mal Respir 42(5):274–285 40263022 10.1016/j.rmr.2025.03.003

[22] Swain SM, Nishino M, Lancaster LH, Li BT, Nicholson AG, Bartholmai BJ, et al. 2022 Multidisciplinary clinical guidance on trastuzumab deruxtecan (T-DXd)–related interstitial lung disease/pneumonitis—Focus on proactive monitoring, diagnosis, and management. Cancer Treat Rev [Internet]. [cited 2025 Aug 25];106. Available from: https://www.cancertreatmentreviews.com/article/S0305-7372%2822%2900042-1/fulltext 10.1016/j.ctrv.2022.102378 35430509

[23] Lee EM, Jiménez-Fonseca P, Galán-Moral R, Coca-Membribes S, Fernández-Montes A, Sorribes E et al (2023) Toxicities and quality of life during cancer treatment in advanced solid tumors. Curr Oncol 30(10):9205–9216 37887565 10.3390/curroncol30100665 PMC10605504

[24] Sibeoni J, Picard C, Orri M, Labey M, Bousquet G, Verneuil L et al (2018) Patients’ quality of life during active cancer treatment: a qualitative study. BMC Cancer 18(1):951 30286733 10.1186/s12885-018-4868-6 PMC6172766

[25] Leonard R, Hennessy BT, Blum JL, O’Shaughnessy J (2011) Dose-adjusting capecitabine minimizes adverse effects while maintaining efficacy: a retrospective review of capecitabine for metastatic breast cancer. Clin Breast Cancer 11(6):349–356 21856245 10.1016/j.clbc.2011.06.005

[26] Yamamoto D, Sato N, Rai Y, Yamamoto Y, Saito M, Iwata H et al (2017) Efficacy and safety of low-dose capecitabine plus docetaxel versus single-agent docetaxel in patients with anthracycline-pretreated HER2-negative metastatic breast cancer: results from the randomized phase III JO21095 trial. Breast Cancer Res Treat 161(3):473–482 28005247 10.1007/s10549-016-4075-6

[27] Stabellini N, Cullen J, Bittencourt MS, Moore JX, Sutton A, Nain P et al (2024) Allostatic Load/Chronic stress and cardiovascular outcomes in patients diagnosed with breast, lung, or colorectal cancer. J Am Heart Assoc 13(14):e033295 38979791 10.1161/JAHA.123.033295 PMC11292743

[28] Stabellini N, Dmukauskas M, Bittencourt MS, Cullen J, Barda AJ, Moore JX et al (2023) Social determinants of health and racial disparities in cardiac events in breast cancer. J Natl Compr Canc Netw 21(7):705-714.e17 37433439 10.6004/jnccn.2023.7023

[29] $name [Internet]. [cited 2025 Aug 25]. Available from: https://www.uhgiving.org/publications/annual-report

[30] R: The R Project for Statistical Computing [Internet]. [cited 2025 Aug 25]. Available from: https://www.r-project.org/

[31] Posit [Internet]. [cited 2025 Aug 25]. Posit. Available from: https://www.posit.co/

[32] Schmid P, Cortes J, Pusztai L, McArthur H, Kümmel S, Bergh J et al (2020) Pembrolizumab for early triple-negative breast cancer. N Engl J Med 382(9):810–821 32101663 10.1056/NEJMoa1910549

[33] Loeser A, Kim JS, Peppercorn J, Burkard ME, Niemierko A, Juric D et al (2024) The right dose: results of a patient advocate-led survey of individuals with metastatic breast cancer regarding treatment-related side effects and views about dosage assessment to optimize quality of life. JCO Oncol Pract 20(7):972–983 38518184 10.1200/OP.23.00539

[34] Jimenez RB, Schenkel C, Levit LA, Hu B, Lei XJ, Harvey RD et al (2022) Oncologists’ perspectives on individualizing dose selection for patients with metastatic cancer. JCO Oncol Pract 18(11):e1807–e1817 36126244 10.1200/OP.22.00427

[35] Commissioner O of the. Project optimus. FDA [Internet]. 2024 Dec 6 [cited 2025 Sept 25]; Available from: https://www.fda.gov/about-fda/oncology-center-excellence/project-optimus

[36] Sledge GW, Neuberg D, Bernardo P, Ingle JN, Martino S, Rowinsky EK et al (2003) Phase III trial of doxorubicin, paclitaxel, and the combination of doxorubicin and paclitaxel as front-line chemotherapy for metastatic breast cancer: an intergroup trial (E1193). J Clin Oncol Off J Am Soc Clin Oncol 21(4):588–592 10.1200/JCO.2003.08.013 12586793

[37] Levit LA, Shah M, Ratain MJ, Garrett-Mayer E, Rahman A, Theoret M et al (2025) Totality of the evidence: optimizing dosage selection strategies in oncology. J Clin Oncol 43(25):2827–2833 40706003 10.1200/JCO-25-00488 PMC12313202

[38] Wang R, Hu B, Pan Z, Mo C, Zhao X, Liu G et al (2025) Antibody-Drug Conjugates (ADCs): current and future biopharmaceuticals. J Hematol OncolJ Hematol Oncol 18(1):51 40307936 10.1186/s13045-025-01704-3 PMC12044742

[39] Hu Q, Wang L, Yang Y, Lee JB (2024) Review of dose justifications for antibody-drug conjugate approvals from clinical pharmacology perspective: a focus on exposure-response analyses. J Pharm Sci 113(12):3434–3446 39374692 10.1016/j.xphs.2024.10.002

[40] Research C for DE and. Clinical pharmacology considerations for antibody-drug conjugates guidance for industry [Internet]. FDA; 2024 [cited 2025 Aug 26]. Available from: https://www.fda.gov/regulatory-information/search-fda-guidance-documents/clinical-pharmacology-considerations-antibody-drug-conjugates-guidance-industry

[41] Gerber HP, Gangwar S, Betts A (2023) Therapeutic index improvement of antibody-drug conjugates. MAbs 15(1):2230618 37408311 10.1080/19420862.2023.2230618 PMC10324427

[42] Murphy R, Halford S, Symeonides SN (2023) Project optimus, an FDA initiative: considerations for cancer drug development internationally, from an academic perspective. Front Oncol 3(13):1144056 10.3389/fonc.2023.1144056 PMC10020863 36937434

[43] Rüsing LZ, Schweighofer J, Aschauer J, Jeryczynski G, Vospernik L, Gisslinger H et al (2025) Optimizing Belantamab Mafodotin in relapsed or refractory multiple myeloma: impact of dose modifications on adverse events and hematologic response in a real-world retrospective study. Cancers 17(14):2398 40723281 10.3390/cancers17142398 PMC12294064

[44] Lee HY, Shih V, Chan JJ, Liong SZ, Tan RSYC, Ma J et al (2025) Evaluating the impact of relative dose intensity on efficacy of trastuzumab deruxtecan for metastatic breast cancer in the real-world clinical setting. Ann Acad Med Singapore 54(8):458–466 40928858 10.47102/annals-acadmedsg.202576

[45] Chow RD, Sedhom R, Mamtani R. Reduced-dose enfortumab vedotin, treatment continuity, and survival in urothelial cancer. JAMA Oncol [Internet]. 2025 Nov 13 [cited 2025 Dec 20]; Available from: 10.1001/jamaoncol.2025.4566 10.1001/jamaoncol.2025.4566 PMC12616528 41231504

[46] Tang E, Rowland A, McKinnon RA, Sorich MJ, Hopkins AM (2019) Effect of early adverse events resulting in ado-trastuzumab emtansine dose adjustments on survival outcomes of HER2+ advanced breast cancer patients. Breast Cancer Res Treat 178(2):473–477 31399933 10.1007/s10549-019-05393-8

[47] Rugo HS, Tolaney SM, Loirat D, Punie K, Bardia A, Hurvitz SA et al (2022) Safety analyses from the phase 3 ASCENT trial of sacituzumab govitecan in metastatic triple-negative breast cancer. Npj Breast Cancer 8(1):98 36038616 10.1038/s41523-022-00467-1 PMC9424318

[48] Pérez García JM, Gión M, Ruiz-Borrego M, Blancas I, Lopez-Miranda E, Blanch S et al (2024) Prevention of sacituzumab govitecan (SG)-related neutropenia and diarrhea in patients (pts) with triple-negative or HR+/HER2- advanced breast cancer (ABC; PRIMED): a phase 2 trial. J Clin Oncol 42(16_suppl):1101–1101

[49] Pérez-García JM, Gion M, Ruiz-Borrego M, Blancas I, López-Miranda E, Blanch S, et al. 2025 Prevention of sacituzumab govitecan-related neutropenia and diarrhea in patients with HER2-negative advanced breast cancer (PRIMED): an open-label, single-arm, phase 2 trial. eClinicalMedicine [Internet]. [cited 2025 Sept 25];85. Available from: https://www.thelancet.com/journals/eclinm/article/PIIS2589-5370(25)00241-X/fulltext 10.1016/j.eclinm.2025.103309 PMC12221278 40606525

